# Chronic mental health vulnerability: urgent need of transdiagnostic evidence-based interventions

**DOI:** 10.47626/2237-6089-2023-0783

**Published:** 2025-01-07

**Authors:** Luciano Dias de Mattos Souza

**Affiliations:** 1 Universidade Católica de Pelotas Pelotas RS Brazil Universidade Católica de Pelotas (UCPel), Pelotas, RS, Brazil.

The recent study on the incidence of suicidal ideation among the Brazilian Longitudinal Study of Adult Health (Estudo Longitudinal de Saúde do Adulto [ELSA-Brasil]) cohort participants^1^ illuminates the profound impacts of the coronavirus disease 2019 (COVID-19) pandemic on mental health. The findings indicate a heightened likelihood of suicidal ideation in individuals with a history of adverse childhood experiences and common mental disorders. This alarming trend, in conjunction with the theoretical understanding of suicidal behavior,^2^ points to a significant segment of the population developing chronic mental health vulnerabilities. In stressful environments or situations, these individuals are predisposed to significant behavioral dysfunctions. This revelation underscores the urgent necessity for the mental health professional community to adapt and broadly apply evidence-based clinical interventions. Further, there is also an imperative to upscale training for public health professionals, equipping them with the skills to comprehend and implement these interventions effectively.

The challenge lies in translating evidence-based practices into widely accessible interventions while ensuring continuous monitoring of their effectiveness. Mental health professionals must collaboratively strive to modify clinical interventions for large-scale implementation through Brazilian Unified Health System (SUS). These interventions should be customized to address the diverse needs of the population, recognizing the complex nature of mental health issues stemming from varied socioeconomic backgrounds and personal histories. Cognitive-behavioral interventions, such as synchronous online cognitive behavioral therapy (CBT) treatments and mobile mental health applications featuring psychoeducation and self-monitoring tools,^3-5^ show promise in bridging the accessibility gap and are also effective in a transdiagnostic perspective. They enable broader reach and real-time efficacy monitoring.

Highlighting the necessity for comprehensive training of mental health public servants is paramount. Training programs should incorporate philosophical elements of CBT and evidence-based practice^6^ and emphasize understanding the psychosocial context of mental health.^7^ Establishing a robust framework for the continuous education of mental health professionals is essential to maintain their expertise in evidence-based practices.

Despite consistent advances in psychiatric reform, the structure of mental health care services in Brazil still exhibits significant gaps. The limited number of specialized multiprofessional outpatient clinics (AMENT) in Brazil is a case in point.^8^

Consequently, a large segment of the population remains without access to mental health care, as they neither benefit from ongoing primary care mental health services – basic health units (Unidades Básicas de Saúde [UBSs]) –, as nor meet the non-standard criteria for admission to high-complexity care services – like center for psychosocial care (Centro de Atenção Psicossocial [CAPS]). Many of these individuals, as indicated by the ELSA-Brasil cohort, carry risk factors associated with an increased risk of suicide.^1^

While political efforts in recent decades have emphasized the recognition of mental health care as a fundamental human right, numerous barriers still impede access to effective treatment. Future strategies should focus on adapting and validating treatment protocols for common mental disorders in a transdiagnostic perspective within the Brazilian context. Expanding and training secondary care services could alleviate the issues associated with chronic mental health vulnerability.
